# Furocoumarin Content of Fennel—Below the Safety Threshold

**DOI:** 10.3390/molecules24152844

**Published:** 2019-08-05

**Authors:** Diána Kerekes, Attila Csorba, Beáta Gosztola, Éva Németh-Zámbori, Tivadar Kiss, Dezső Csupor

**Affiliations:** 1 Department of Pharmacognosy, University of Szeged, 6720 Szeged, Hungary; 2 Interdisciplinary Centre for Natural Products, University of Szeged, 6720 Szeged, Hungary; 3 Department of Medicinal and Aromatic Plants, Szent István University, 1118 Budapest, Hungary

**Keywords:** fennel, *Foeniculum vulgare*, furocoumarin, psoralene, 5-methoxypsoralene, imperatorin

## Abstract

Furocoumarins are known for their phototoxic and potential carcinogenic effects. These types of compounds have previously been reported from fennel (*Foeniculum vulgare* Mill.), a widely used medicinal plant and spice; however, no reliable quantitative data are available on the occurrence of these compounds in fennel fruits. For the first time, we report a comprehensive analysis of fennel fruit samples of different origins, representing a wide range of accessions for their furocoumarin content. Psoralene, 5-methoxypsoralene (bergapten), and imperatorin contents of 33 fennel samples were analyzed using a sensitive liquid chromatography-mass spectrometry (LC-MS) method. When applied at the highest therapeutic dose described in the monograph issued by the European Medicines Agency, the furocoumarin content of the fruits ranged up to 1.22 μg/d, which is below the most restrictive recommendations. Based on our findings, fennel consumption can be considered as safe, at least based on its low furocoumarin content.

## 1. Introduction

Fennel (*Foeniculum vulgare* Mill.) is a medicinal and aromatic plant cultivated worldwide. Its fruit is used for flavouring (i.e., as a spice) and also as a medicine to alleviate digestive symptoms. In the European official medicine, two varieties, namely *F. vulgare* Miller subsp. *vulgare* var. *vulgare* (bitter fennel) and *F. vulgare* Miller subsp. *vulgare* var. *dulce* (Miller) Thellung. (sweet fennel), are used. These may be applied as traditional herbal medicinal products to alleviate mild, spasmodic gastrointestinal complaints as well as minor spasm associated with menstrual periods, and they are also used as expectorants for coughs associated with common colds. The maximal daily doses range from 2 g (powdered herbal substance) to 7.5 g (as herbal tea) [1,2]. The taste, odor, and pharmacological effects of fennel are related to its essential oil content. The composition of the essential oil is variable, the main components being trans-anethole, fenchone, methylchavicol, limonene, and estragole [3]. Although trans-anethole may trigger hypersensitivity [4], more concern is related to the estragole content of fennel. This compound may be present in a concentration as high as 5% in bitter fennel and 10% in sweet fennel essential oils [5], and it is claimed to have genotoxic and carcinogenic effects [6]. It is questionable, however, whether this hypothetic risk poses real danger in the case of human application. The European Food Safety Authority (EFSA) proposed a risk assessment tool, called margin of exposure (MOE), to assess the risks associated with compounds that are both genotoxic and carcinogenic [7]. According to the Scientific Committee of EFSA, a MOE of 10,000 or higher, if it is based on the BMDL_10_ (benchmark dose resulting in 10% increase of risk) from an animal study, would be of low concern from a public health point of view [8]. Considering the estragole contents of teas prepared from fennel fruits (average concentration 639 µg/L, but in some samples it may reach 4644 µg/L), and with regard to the fact that the calculated MOE values for some samples were below 10,000, the potential risk for human health cannot be excluded [9]. However, fennel tea is usually consumed for short periods of time; therefore, the potential risk might be overestimated. 

Another group of compounds that may pose a risk to fennel consumers include furocoumarins. These compounds are widespread in the plant kingdom but are characteristic to some families, such as Apiaceae. Furocoumarins are carcinogenic in combination with UV light exposure [10]. Cohort studies indicate that the consumption of furocoumarin-rich food may increase the risk of melanoma as well as that of basal and squamous cell carcinoma [11,12]. The photosensitizing activity of furocoumarins is explained by their photochemicals binding to pyrimidine bases of the DNA, resulting in impaired DNA transcription and replication [13]. Crosslink formation is associated with carcinogenicity [14], since DNA crosslink repair processes may generate point mutations, translocations, and deletions, leading to genetic instability [15]. Cross-linking frequently occurs with linear furocoumarins but is uncommon with angular furocoumarins [16].

In the westernized populations, the estimated average total intake of dietary furocoumarins is about 1.2–1.5 mg/d with *Citrus* species, such as grapefruit, being the main sources [16], while high-exposure peak values may reach 14 mg/d [17]. The major furocoumarins of grapefruit are bergaptol, bergamottin, and 6′,7′-dihydroxy-bergamottin [18]. As most of the toxicological studies focus on 5-methoxypsoralen and 8-methoxypsoralen, little is known about the toxicological characteristics of other furocoumarins. Currently, the risk of dietary furocoumarins is considered to be very small or insignificant. Similar to estragole, the MOE approach should be used for the risk assessment of furocoumarins too [7]. However, according to a reflection paper of the European Medicines Agency, the MOE approach would lead to a very low acceptable exposure to furocoumarins, and it is not considered to be an appropriate tool for herbal medicinal products that contain furocoumarins. For total furocoumarins contained in an herbal medicinal preparation, a daily intake of ≤15 μg is considered to pose no unacceptable risks based on the thresholds for toxicological concern, while according to a different approach (comparison with dietary exposure), 1.5 mg of furocoumarins included in herbal medicinal products is not considered to contribute significantly to the overall risk. However, if the latter limit is exceeded, a detailed benefit/risk assessment is necessary [19].

These types of compounds have been detected in several Apicaeae species, such as carrot, parsnip, lovage, parsley, and celery, with psoralen, 5-methoxypsoralen (bergapten), 8-methoxypsoralen, isopimpinellin, oxypeucedanin, and imperatorin being the main constituents [20,21,22,23,24]. The stem base of fennel was reported to contain 5.24 mg/kg of 5-methoxyspsoralen and 2.8 mg/kg of isoimperatorin [22]. From the stems of the plant bergapten, imperatorin and psolaren were isolated (70, 10, and 35 mg from 600 g of dry stem, respectively) [25]. From fennel fruits, 5-methoxypsoralen was isolated by two research groups (concentration not known) [26,27]. An LC-MS based study did not report the presence of furocoumarins in fennel teas; however, the identification of this type of compounds was out of the focus of the research [28]. 

Based on the phytochemical studies carried out with different parts of fennel, the risk associated with the furocoumarin content of fennel fruits cannot be assessed due to the lack of quantitative data. Fennel fruits, most frequently consumed as tea, are widely used to alleviate various gastrointestinal symptoms, even in children and infants, and is also used as a galactogogue [29]. Considering the theoretically possible risks posed by the consumption of this furocoumarin-containing plant, the aim of our study was to quantitively measure the furocoumarin content of different fennel fruit samples in order to provide appropriate data for risk assessment.

## 2. Results

**Figure 1:**
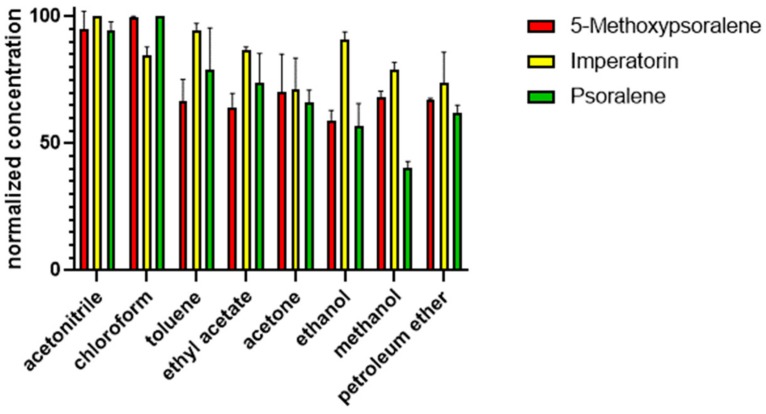
Extraction efficiencies of different solvents expressed as the normalized concentrations of the analytes.

Our experiments have focused on three furocoumarins, namely psoralene, 5-methoxypsoralene, and imperatorin, reported in previous papers to be present in different parts of fennel. Extraction optimization revealed that acetonitrile was the most appropriate solvent to extract the maximal amounts of these three furocoumarins analyzed (Figure 1). Thus, our experiments were carried out using acetonitrile. These experiments revealed no remarkable differences in recovery values between samples spiked before and after extraction, suggesting that the analytes were stable during the extraction, and the relatively high (80.17%–133.44%) recovery values confirmed the validity of the sample preparation procedure (Table 1). 

**Figure 2:**
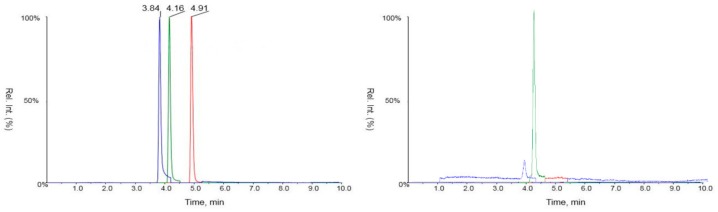
LC-MS chromatograms of the mixture of psoralene (R_t_ = 3.84 min), 5-methoxypsoralene (R_t_ = 4.16 min), and imperatorin (R_t_ = 4.91 min) (left) and of a fennel extract (right).

Psoralene, 5-methoxypsoralene, and imperatorin could be detected with good resolution and selectivity in the applied analytical system (Figure 2). The values for the limit of detection and the limit of quantification were 8.8–10.1 and 28.7–31.3 nM, respectively. Linear calibration curves of these compounds were established in the concentration range of 10–1000 nM, characterized by the equations *y* = 12.2*x* + 185 (R^2^ = 0.9960), *y* = 38*x* + 159 (R^2^ = 0.9991), and *y* = 96.3*x* − 1.25 × 10^−3^ (R^2^ = 0.9997) for psoralene, 5-methoxypsoralene, and imperatorin, respectively. Intraday precision ranged from 0.67% to 1.81% for the reference standards (RSD%, *n* = 12). These results underline the good reproducibility of our method.

The level of imperatorin was below the limit of detection in all analyzed samples. Psoralene could not be detected in 19 samples, whereas 5-methoxypsoralene could not be detected in 7 samples (Table 2). The concentration of psoralene was approximately one order of magnitude lower than that of 5-methoxypsoralene. The amounts of psoralene and 5-methoxypsoralene were calculated for the maximal therapeutic dose (7.5 g) of fennel according to the monographs of the European Medicines Agency [1,2]. In 7 of the 33 samples, the amount of total furocoumarins was below the limit of detection. Furocoumarin-containing samples were characterized by total furocoumarin contents in the range of 0.0099–1.2209 µg/7.5 g.

## 3. Discussion

Considering that the average daily dietary intake of fennel is in the range of 1.2–1.5 mg, and with regard to the fact that according to the reflection paper of the European Medicines Agency daily intake of ≤15 μg of furocoumarins in an herbal medicinal preparation does not pose any unacceptable risk [19], the therapeutic use of fennel seems to be safe, at least for its furocoumarin content, since all the samples we examined were below this limit. Moreover, since the values presented here were determined from extracts prepared with an organic solvent to achieve maximal extraction, it can be supposed that the furocoumarin content of herbal infusions prepared with water, a less optimal extracting solvent, might be even lower.

## 4. Materials and Methods 

### 4.1. Plant Material

Ripe fruits of 33 *F. vulgare* Mill. subsp. *vulgare* var. *vulgare* accessions were used for the analysis, all acquired from the collection of the Genebank of the Department of Medicinal and Aromatic Plants, Szent István University, Hungary. Among the examined bitter fennel accessions there were cultivated and wild growing populations of unknown origin (samples 1–25), nonrelated progenies of former breeding work (samples 26–30), and three registered varieties (Berfena, Groβfrüchtig, and Soroksári). For comparison, a caraway commercial fruit sample was also investigated.

### 4.2. Extraction

To optimize extraction by choosing the most efficient extracting solvent, methanol, ethanol, petroleum ether, chloroform, acetonitrile, acetone, toluene, and ethyl acetate were applied for extraction. All solvents were of HPLC gradient grade quantity (VWR International LLC, Radnor, PA USA). A total of 0.500 g of the freshly ground dry fruits was extracted with 4.95 ml extracting solvent for 10 min in an ultrasonic bath, and then it was spiked with 0.05 mL of a solution containing psoralene, 5-methoxypsoralene, and imperatorin in 100 μM concentrations each. In this experiment, furocoumarins were added to the samples, since preliminary tests referred to very low quantities of these compounds. The extracts were filtered through 0.45 μM polytetrafluoroethylene (PTFE) syringe filters. A total of 3.00 mL of the extracts were evaporated to dryness under nitrogen stream, re-dissolved in 1 mL methanol:water 3:1, and were filtered again. 

To evaluate whether sample preparation affected quantitative determination, two experiments were carried out. First, 0.500 g plant material was spiked with 10 μL stock solution containing known amounts of psoralene, 5-methoxypsoralene, and imperatorin, and then 4.99 mL acetonitrile was added. Extraction was performed as described above. In the second experiment, the sample was spiked after extraction and filtering. One sample was processed the same way but without spiking. 

### 4.3. Determination and Quantification of Furocoumarins in Fennel Extracts by LC-MS

Plant samples were extracted, as described in the extraction section, using acetonitrile (5.00 for 0.5000 mg plant material) as the extracting solvent. For separation, a Kinetex^®^ 2.6 µm C18 100 Å, 100 × 2.1 mm column was used, with water (A) and acetonitrile (B) as eluents (HPLC gradient grade, VWR International Ltd.) both containing 0.1% formic acid. The flow was 0.5 mL/min throughout the separation process, and the column temperature was set to 40 °C. The ratio of B increased from 5% to 95% within 5 min, followed by washing and equilibrating phases (both 2.5 min). Analysis was carried out on a Shimadzu HPLC system (Shimadzu Corporation, Kyoto, Japan) comprising an LC-20AD pump, a DGU-20A5R degasser, an SIL-20ACH autosampler (tempered to 21 °C), a CTO-20AC column oven, and SPD-M20A photodiode array detector modules (connected with CBM-20A control module) coupled to an AB Sciex API 2000 triple quadrupole mass spectrometer (AB Sciex LLC, Redwood City, CA, USA). Furocoumarins were detected using the single reaction monitoring mode (SRM). Precursor ions were detected at *m*/*z* values of 187.4, 217.3, and 271.2, whereas product ions were detected at *m*/*z* 131.2, 202.2, and 203.3 in the cases of psoralene, 5-methoxypsoralene, and imperatorin, respectively. Collision energies were 36, 31, and 19 eV, respectively. Calibration curves were established based of the quantifications of six solutions containing the mixtures of the three analytes. The signal-to-noise ratio was used to express the limit of detection (three times the noise) and the limit of quantitation (ten times the noise). Intraday precisions were calculated from data acquired during a three-day validation. Precision was expressed as relative standard deviation (RSD%).

## 5. Conclusions

The results presented here confirm that the therapeutic use of fennel fruits (not exceeding 7.5 g/day) can be considered as safe for adults, at least based on the low furocoumarin content of the plant material. 

## Figures and Tables

**Table 1:** Recovery values of the analytes when added to the sample before and after extraction.

Sample	Recovery (%)		
	Psoralene	5-Methoxypsoralene	Imperatorin
Spiked before extraction	101.34	105.81	84.56
Spiked after extraction	133.44	80.17	92.42

**Table 2:** Furocoumarin contents of fennel fruit samples.

Sample	μg/7.5 g Plant Material (± SD)		
	Psoralene	5-Methoxypsoralene	Total
1	0.0551 ± 0.0015	0.6829 ± 0.0311	0.7380
2	0.0431 ± 0.0017	0.4830 ± 0.0172	0.5261
3	0.0504 ± 0.0011	0.6192 ± 0.0256	0.6696
4	0.0561 ± 0.0019	0.6419 ± 0.0301	0.6980
5	0.0646 ± 0.0031	0.6684 ± 0.0219	0.7330
6	0.0449 ± 0.0011	0.5239 ± 0.0092	0.5688
7	0.1072 ± 0.0050	1.1137 ± 0.0112	1.2209
8	0.0886 ± 0.0039	1.1052 ± 0.0216	1.1938
9	0.0938 ± 0.0045	1.0114 ± 0.0328	1.1052
10	0.0710 ± 0.0018	0.8730 ± 0.0178	0.9440
11	0.0175 ± 0.0006	0.1165 ± 0.0033	0.1340
12	*	0.0220 ± 0.0007	0.0220
13	*	0.0236 ± 0.0009	0.0236
14	*	*	*
15	*	0.0183 ± 0.0008	0.0183
16	0.0142 ± 0.0002	0.0618 ± 0.0021	0.0760
17	*	*	*
18	*	*	*
19	*	*	*
20	0.0193 ± 0.0007	0.2360 ± 0.0072	0.2553
21	*	*	*
22	*	0.0116 ± 0.0003	0.0116
23	*	*	*
24	*	*	*
25	0.0133 ± 0.0005	0.1184 ± 0.0039	0.1317
26	0.0078 ± 0.0002	0.0321 ± 0.0007	0.0399
27	*	0.0188 ± 0.0006	0.0188
28	*	0.0426 ± 0.0018	0.0426
29	*	0.0111 ± 0.0003	0.0111
30	*	0.1045 ± 0.0061	0.1045
variety Berfena	*	0.0634 ± 0.0028	0.0634
variety Groβfrüchtig	*	0.0099 ± 0.0002	0.0099
variety Soroksár’	*	0.0745 ± 0.0011	0.0745
caraway commercial sample	*	0.1062 ± 0.0018	0.1062

* below the level of detection.
